# Religious and Spiritual Beliefs and Practices in Older Survivors of a Heart Attack and Quality of Life

**DOI:** 10.1093/geroni/igaf122.1048

**Published:** 2025-12-31

**Authors:** Hawa Abu, Jerry Gurwitz, Robert Goldberg, David Dosa, Daniel Matlock, barbara Kivowitz, Alok Kapoor, David McManus

**Affiliations:** University of Massachusetts Chan Medical School, Worcester, Massachusetts, United States; University of Massachusetts Chan Medical School, Worcester, Massachusetts, United States; University of Massachusetts Chan Medical School, Worcester, Massachusetts, United States; University of Massachusetts Chan Medical School, Worcester, Massachusetts, United States; University of Colorado Anschutz Medical Campus, Aurora, Colorado, United States; San Francisco, California, United States; University of Massachusetts Chan Medical School, Worcester, Massachusetts, United States; University of Massachusetts Chan Medical School, Worcester, Massachusetts, United States

## Abstract

Religious and spiritual beliefs and practices (R/S) are often used by patients to cope with life-threatening events. However, the role of R/S remains understudied among older adults who survive a heart attack. The study objective was to examine the extent of engagement in R/S among older adults hospitalized for acute coronary syndrome (ACS) and their association with physical health related quality of life (HRQoL). Data was obtained from the multi-center prospective TRACE-CORE study (2011-2013). Patients aged ≥65 years hospitalized with ACS at six medical centers in Massachusetts and Georgia were included in the present study. Participants self-reported their extent of engagement in three items assessing R/S – deriving strength and comfort from religion, making petition prayers for their health, and receiving intercessory prayers. Physical HRQoL was measured with the SF-36®v2 physical component score. Linear regression models were used to examine the relationship between each R/S measure and physical HRQOL during hospitalization. Among 804 older adults hospitalized with ACS (mean age 73 years; 39% women; 87% non-Hispanic White), 88% found strength and comfort from religion, 67% prayed for their health, and 88% received intercessory prayers. The average physical HRQoL score was 40.2. After adjusting for potential confounders, greater engagement in R/S was associated with poorer physical HRQoL during hospitalization. A majority of older survivors of ACS utilize their R/S beliefs as coping mechanisms and their relationship to physical HRQoL is complex. Integrating R/S support into patient-centered care may support recovery and QoL after a heart attack.

